# Ultrahigh-Throughput Multiplexing and Sequencing of >500-Base-Pair Amplicon Regions on the Illumina HiSeq 2500 Platform

**DOI:** 10.1128/mSystems.00029-19

**Published:** 2019-02-19

**Authors:** Johanna B. Holm, Michael S. Humphrys, Courtney K. Robinson, Matthew L. Settles, Sandra Ott, Li Fu, Hongqiu Yang, Pawel Gajer, Xin He, Elias McComb, Patti E. Gravitt, Khalil G. Ghanem, Rebecca M. Brotman, Jacques Ravel

**Affiliations:** a Institute for Genome Sciences and Department of Microbiology and Immunology, University of Maryland School of Medicine, Baltimore, Maryland, USA; b Davis Genome Center, University of California, Davis, California, USA; c Department of Epidemiology and Biostatistics, School of Public Health, University of Maryland, College Park, Maryland, USA; d Milken Institute School of Public Health, George Washington University, Washington, DC, USA; e Department of Medicine, Johns Hopkins School of Medicine, Baltimore, Maryland, USA; University of Waterloo

**Keywords:** 16S RNA, amplicon sequencing, PCR, ultrahigh throughput

## Abstract

Amplification, sequencing, and analysis of the 16S rRNA gene affords characterization of microbial community composition. As this tool has become more popular and amplicon-sequencing applications have grown in the total number of samples, growth in sample multiplexing is becoming necessary while maintaining high sequence quality and sequencing depth. Here, modifications to the Illumina HiSeq 2500 platform are described which produce greater multiplexing capabilities and 300-bp paired-end reads of higher quality than those produced by the current Illumina MiSeq platform. To improve the feasibility and flexibility of this method, a 2-step PCR amplification protocol is also described that allows for targeting of different amplicon regions, and enhances amplification success from samples with low bacterial bioburden.

**IMPORTANCE** Amplicon sequencing has become a popular and widespread tool for surveying microbial communities. Lower overall costs associated with high-throughput sequencing have made it a widely adopted approach, especially for projects that necessitate sample multiplexing to eliminate batch effect and reduced time to acquire data. The method for amplicon sequencing on the Illumina HiSeq 2500 platform described here provides improved multiplexing capabilities while simultaneously producing greater quality sequence data and lower per-sample cost relative to those of the Illumina MiSeq platform without sacrificing amplicon length. To make this method more flexible for various amplicon-targeted regions as well as improve amplification from low-biomass samples, we also present and validate a 2-step PCR library preparation method.

## Abstract (precis)

Amplicon sequencing has become a popular and widespread tool for surveying microbial communities. Lower overall costs associated with high-throughput sequencing have made it a widely adopted approach, especially for projects that necessitate sample multiplexing to eliminate batch effect and reduced time to acquire data. The method for amplicon sequencing on the Illumina HiSeq 2500 platform described here provides improved multiplexing capabilities while simultaneously producing greater quality sequence data and lower per-sample cost relative to those of the Illumina MiSeq platform without sacrificing amplicon length. To make this method more flexible for various amplicon-targeted regions as well as improve amplification from low-biomass samples, we also present and validate a 2-step PCR library preparation method.

## INTRODUCTION

The introduction of the Illumina HiSeq and MiSeq platforms has allowed for the characterization of microbial community composition and structure by enabling in-depth, paired-end sequencing of amplified fragments of the 16S rRNA gene, the internal transcribed spacer (ITS) region, and other marker genes. The Illumina MiSeq instrument produces paired sequence reads up to 300 bp long. However, low amplicon sequence diversity often results in reduced sequence read quality because of the homogenous signals generated across the entire flow cell (1). The cosequencing of PhiX DNA can alleviate the problem, but it reduces the overall sequence read throughput and multiplexing options. Alternatively, the addition of a heterogeneity spacer in the amplification primer offsets the sequence reads by up to 7 bases and simultaneously increases multiplexing capacity by lowering the amount of PhiX control DNA to ∼5% (1). Lower overall costs associated with high-throughput sequencing have made it a widely adopted approach, especially for projects which necessitate sample multiplexing to eliminate batch effect and reduced time to acquire data, which is often the case in sequencing cores. The Illumina HiSeq 2500 platform, with its high throughput, offers a remedy to limitations in multiplexing but can currently only be used on short amplicons (i.e., the 16S rRNA gene V4 region) due to limitations in read length (maximum of 25 bp PE Rapid Run Mode on a HiSeq 2500 instrument) (2).

We present a method that produces high-quality 300-bp paired-end reads (median Q-score, 37.1) from up to 1,568 samples per lane on a HiSeq 2500 instrument set to Rapid Run Mode. To make this method feasible and flexible in sequencing different amplicon regions, libraries are prepared using a modified version of previously published 1-step PCR (1) and 2-step PCR (https://support.illumina.com/downloads/16s_metagenomic_sequencing_library_preparation.html) methods. In the 1-step PCR method, fusion primers that contain both the target amplification primer, the heterogeneity spacer, the barcode, and the sequencing primers have been used to amplify a ready-to-sequence amplicon. However, primers ranging from 90 to 97 bp in length are expensive, can be subject to degradation, are associated with poor or no amplification from low-biomass samples, and are limited to the targeted amplicon region. The 2-step PCR library preparation procedure described here is more flexible and improves amplification from low-biomass samples because the 1st-step primers are short, target the amplicon region of interest, and contain the heterogeneity spacer and Illumina sequencing primer. The barcodes and flow-cell linker sequences are introduced in a second round of PCR by using the Illumina sequencing primer as a target.

**FIG 1:**
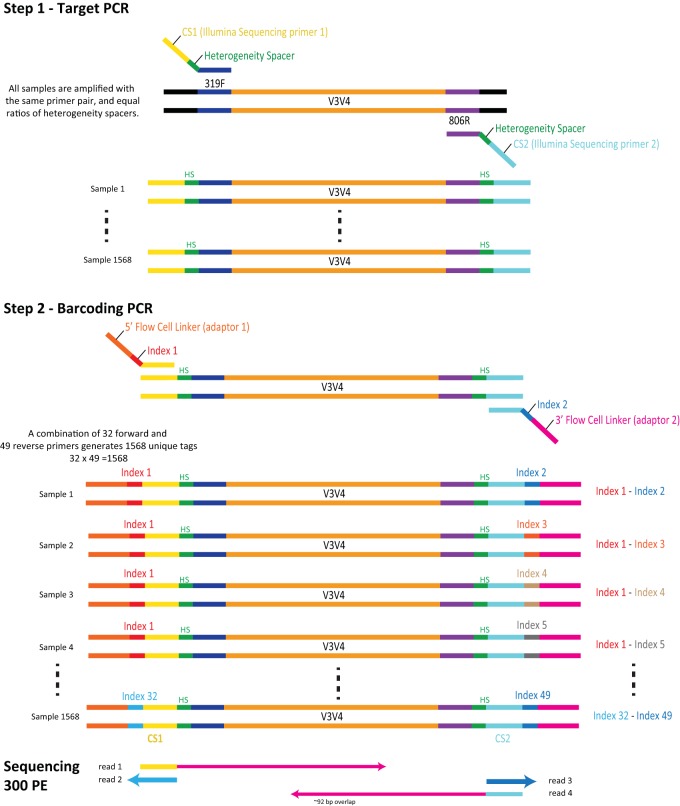
Illumina amplicon library preparation through 2-step PCR amplification. In the step 1 PCR, the target gene is amplified using primers that contain the heterogeneity space and the CS1 Illumina sequencing primers. The 2nd PCR targets the CS1 Illumina sequencing primer to add the indices and the Illumina flow-cell linker sequence. Sequencing proceeds wherein reads 1 and 4 contain the forward and reverse target gene sequence, respectively, and reads 2 and 3 contain the first and second barcode indices, respectively.

A previously published 2-step PCR method (2) used triple barcode indexing, produced 250-bp paired-end reads on the Illumina HiSeq 2500 platform, and reported a taxon-specific sequencing bias of the first-step primers which differed in both barcode sequence and heterogeneity spacer length. The method we present here (Fig. 1) uses 8-bp dual indexing, as described by Fadrosh et al. (1), wherein the forward index is never used as a reverse index, produces 300-bp paired-end reads by modifying the Illumina HiSeq 2500 sequencing method, and attempts to control for amplification biases by (i) implementing an equimolar ratio of all PCR step 1 primers (which differ only in the length of the heterogeneity spacers) provided to each sample to reduce biases imposed by the heterogeneity spacer, and (ii) introducing barcode sequences in the second PCR step of the library preparation.

In addition to the benefit of flexibility in choice of gene target, we show that the 2-step PCR method improves amplification success of low-biomass samples relative to that of the 1-step PCR method. Additionally, we show that the 2-step PCR method does not significantly bias the measured microbial community by comparing vaginal community state types (3) as defined by taxonomic profiling of vaginal samples of pre- and postmenopausal women (4) targeting the V3-V4 region of the 16S rRNA gene. Postmenopausal vaginal samples tend to have lower absolute bacterial load relative to that of premenopausal samples (5, 6), making amplification challenging. Samples from each woman were prepared using the 1-step PCR procedure (1) sequenced on the Illumina MiSeq platform and the 2-step PCR procedure sequenced on both the Illumina MiSeq and HiSeq platforms. In addition to comparing the quality of libraries sequenced on the Illumina HiSeq and MiSeq platforms, we also sought to measure (i) improved amplification efficiency of samples prepared by the 2-step PCR method compared to the 1-step method and (ii) the differences in intraindividual vaginal community state types between methods. Finally, we demonstrate the precision of this method using a comparative mock community analysis.

## RESULTS

### 2-Step PCR amplicon library preparation improves amplification success of low-biomass vaginal samples.

Amplification failure was more common in the 1-step PCR amplification protocol than the 2-step PCR method presented here (Fig. 1) due to the long primers which degrade over time, thereby reducing amplification efficiency, especially for low-biomass samples (i.e., low absolute bacterial load). Figure S2 in the supplemental material contains an example electrophoresis gel labeled with the volume of the library used for pooling. Samples labeled 20 show no bands and, in this analysis, represent a failure of amplification. All other samples represent successful amplifications. Of 92 low-biomass vaginal samples (mean subject age, 48.9 years), 54% were successfully amplified using the 1-step PCR protocol, while the 2-step protocol produced amplifications from 90% of samples (Table 1). Of 42 vaginal samples that did not amplify by the 1-step method, 55% were from women over the age of 51, the average age of menopause. Thirty-four of these samples were successfully amplified using the 2-step method, an 80% improvement (Data Set S1). A panbacterial quantitative PCR (qPCR) analysis confirmed the significantly lower number of 16S rRNA gene targets in the samples that were amplified by 2-step PCR but not 1-step PCR (*U* = 790.5, *P* = 0.03) (Fig. S5). Subjects in this group were also significantly older (*U* = 484, *P* = 0.001). Amplicons were not observed from 8 samples regardless of protocol type, and 1 sample was successfully amplified using the 1-step but not the 2-step procedure.

**TABLE 1:** Summary of sequencing results for vaginal samples^*a*^

Library preparation method parameter	Result by PCR type		
	1-step	2-step	2-step
No. of samples subjected to amplification	92	92	92
No. of samples successfully amplified	49	83	83
Sequencing platform	MiSeq	MiSeq	HiSeq
Chimeric sequences detected (%)	0.70	3.3	3.1
Mean no. (±SE) of nonchimeric, assembled sequences per sample	11,080 ± 1,506	14,282 ± 483	50,514 ± 4,427
Median quality score per sample (Q1–Q3)	36.2^*b*^ (33.5–37.2)	34.9^*b*^ (29.9–36.3)	37.1^*b*^ (33.0–38.0)

a Figure S4 summarizes the prequality filtering per-cycle quality scores.

b Significant. Kruskal-Wallis, H = 187.85; P < 2.2 × 10^−16^.

10.1128/mSystems.00029-19.10 DATA SET S1 Table S1a shows 2-step oligonucleotides for step 1, Table S1b shows per-sample dual-index strategy for a full HiSeq run (17 plates), Table S1c shows 2-step forward adapter oligonucleotides for step 2, and Table S1d shows 2-step reverse adapter oligonucleotides for step 2. Table S2 shows amplification success of low-biomass samples via 1-step and 2-step PCR library preparation methods. Table S3 shows raw read count table for samples prepared by either 1-step MiSeq, 2-step Miseq, or 2-step HiSeq library preparation and sequencing methods. Table S4 shows raw read count table for Zymo control DNA prepared by 2-step PCR and sequenced by HiSeq or MiSeq sequencing methods. Download Data Set S1, XLS file, 0.6 MB. Copyright © 2019 Holm et al. 2019 Holm et al. This content is distributed under the terms of the Creative Commons Attribution 4.0 International license.

### Samples successfully amplified by both 1-step and 2-step library preparation methods yield similar sequencing metrics on both Illumina MiSeq and HiSeq platforms.

**FIG 2:**
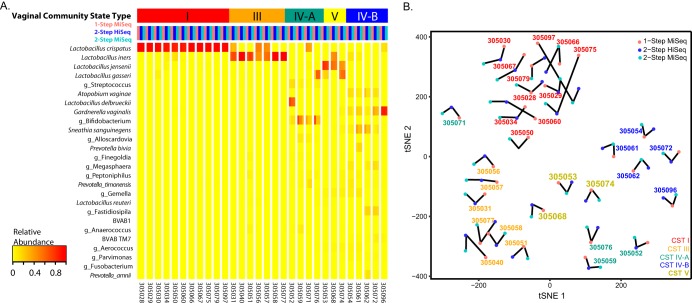
Heatmap of taxon relative abundances (rows) of samples (columns). Subject samples are separated by white lines, and samples are ordered by vaginal community state types and with the following color code: 1-step MiSeq, pink; 2-step HiSeq, blue; 2-step MiSeq, aqua. (B) tSNE representation of Jensen-Shannon distances between samples from the same subject. Samples primarily cluster by vaginal CST.

Samples successfully amplified using both library preparation methods (*n* = 49) were used to compare the 1-step and 2-step library preparation methods sequenced on the Illumina HiSeq (2-step only) and MiSeq (1-step and 2-step) platforms. From each combination of methods, 0.7 to 3% of sequences were detected as chimeras and removed. This yielded, on average, 11,080 sequences per sample from the 1-step library sequenced on the MiSeq platform, 14,282 sequences per sample from the 2-step library sequenced on the MiSeq platform, and 50,514 sequences per sample from the 2-step library sequenced on the HiSeq platform (Table 1). Due to low total read counts from some samples, only 30 samples containing >500 total sequences in each method were used for comparative β-diversity analysis between the three methods. Consistency of observed vaginal community state types (CSTs) between libraries was tested using Fleiss’ kappa for interrater reliability, where κ of >0.75 indicated excellent agreement. Complete agreement between all three methods was observed and samples clustered primarily by vaginal community state type and subject, as opposed to library preparation method or sequencing platform (κ = 1.0) (Fig. 2; raw read count taxonomy tables are available in Data Set S1).

### Mock community libraries prepared via 2-step PCR and sequenced on Illumina HiSeq are not different from those sequenced on Illumina MiSeq.

In order to verify that consistency of results was not simply due to sample type, we also compared the microbial compositions of the ZymoBIOMICS microbial DNA standard obtained on the Illumina HiSeq and MiSeq platforms. We used theoretical values reported by Zymo Research as well as compositional data produced from 16S rRNA gene V3-V4 region amplicon libraries sequenced on the Illumina MiSeq (prepared, sequenced, and provided by Zymo). The raw read count taxonomy table is available in Data Set S1. The distribution of Jensen-Shannon distances between Zymo-prepared, MiSeq-sequenced microbiota composition and theoretical composition did not significantly differ from the distribution of distances between the 2-step-prepared, Illumina HiSeq-sequenced, and theoretical microbiota compositions (*U* = 29, P = 0.9578) (Fig. S6).

### Comparison of Illumina MiSeq and Illumina HiSeq amplicon sequence read quality and quantity.

**FIG 3:**
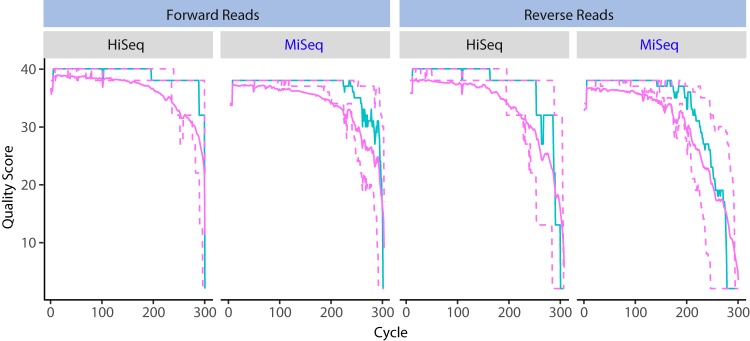
Forward and reverse read quality profiles for 300 cycles on the Illumina HiSeq (1,536 samples) and MiSeq (444 samples) platforms. Amplicon libraries were prepared using a 2-step PCR method. Shown for each cycle are the mean quality score (green line), the median quality score (solid purple line), and the quartiles of the quality score distribution (dotted purple lines).

To compare the quality of amplicon sequence reads produced via 2-step PCR and the Illumina MiSeq and HiSeq platforms, each sequencing run was demultiplexed with the same mapping file, and the sequence read quality profiles were compared. The two runs had 183 samples in common. Significantly greater mean quality scores of both forward and reverse reads were observed for 1,194 samples run on the HiSeq platform compared to 276 samples run on the MiSeq platform (*P* < 2.2 × 10^−16^) (Fig. 3). The HiSeq 2500 platform produced a greater mean number of quality-filtered sequences per sample than the MiSeq platform, with fewer chimeric sequences detected on average (Table 2). These results were also consistent across multiple sequencing runs (Fig. S7). Additionally, the HiSeq 2500 sequencing strategy was more cost efficient (nearly 40% less expensive per sample), assuming 2 lanes are run with 1,568 multiplexed samples per lane (Table 2).

**TABLE 2:** Sequencing run information for the MiSeq and HiSeq platforms

Sequencing platform parameter	MiSeq	HiSeq2500 RR
Run details	2× 300 bp PE	2× 250 bp PE + 2× 50 bp PE
Mean no. (±SE) of assembled sequences per sample	13,116 ± 479	49,851± 895
No. of samples	276	1,194
Chimeric sequences detected (%)	2.8	7.7
Mean no. (±SE) of nonchimeric, assembled sequences per sample	12,737 ± 463	45,988 ± 787
Median quality score (Q25–Q75), forward reads	35.7^*a*^ (33–37)	36.1^*a*^ (35–38)
Median quality score (Q25–Q75), reverse reads	33.9^*b*^ (25–36)	33.2^*b*^ (31–37)
Cost of sequencing per sample (no. of multiplexed samples)	$6.38 (384)	$3.99 (1,568)

a Significant. Wilcoxon rank sum, *W* = 70352; *P* < 2.2 × 10^−16^.

b Significant. Wilcoxon rank sum, *W* = 76453; *P* < 2.2 × 10^−16^.

## DISCUSSION

Microbiome analyses large enough to achieve adequate statistical power are becoming more desirable, and reduced sequencing costs make these analyses feasible. Therefore, ultrahigh-throughput sequencing capabilities are needed that do not sacrifice sequence quality. Ideally, such methods would allow for flexibility to target a diverse set of genes or gene regions (for example, ITS regions, the 16S and 23S rRNA genes, and the *cpn60* gene [7, 8], among others) while also maintaining the ability to sequence longer amplicons (i.e., the 16S rRNA gene V3-V4 region). The method presented here improves on current technologies by producing consistent high-quality, 300-bp paired-end reads. Relative to the Illumina MiSeq platform, sequencing on the Illumina HiSeq platform produced a greater number of reads per sample, of significantly higher quality, with the capability to multiplex up to 2× 1,568 samples. The innovative use of the Illumina HiSeq 2500 platform as presented here and by de Muinck et al. (2) allows for ultrahigh-throughput sequencing of amplicon libraries.

In addition, the 2-step PCR library preparation method described here makes production of sequencing libraries from various gene targets and samples containing low bacterial loads easy through the use of unindexed, target-specific primers in the first round of PCR. Amplification success of samples with low bacterial loads are prone to amplification difficulties, and amplification using the longer primers required in the traditional 1-step protocol (1) exacerbate the problem because of primer degradation and poor annealing due to the long-overhang unprimed sequence. Using the 2-step PCR approach, we showed an 80% improvement over the 1-step PCR method for samples containing low bacterial loads. In addition, the shorter primers used in the 2-step PCR library protocol do not require PAGE purification, lowering the overall cost of the method relative to that of the 1-step PCR protocol. Other low-biomass environments that could benefit from this 2-step PCR procedure include blood and serum (9), respiratory airways (10), skin (11), subseafloor sediments (12), and clean rooms (13).

In summary, to demonstrate the comparability of sequence data sets produced via different methods, 16S rRNA gene V3-V4 region sequence data sets were generated from low-biomass vaginal samples from women using both 1-step and 2-step PCR library construction methods and the Illumina HiSeq and MiSeq sequencing platforms. Complete within-subject agreement between the vaginal community state type assignments (3) were observed between all three methods, although a greater number of significantly higher quality sequences were obtained from the 2-step PCR method sequenced on the Illumina HiSeq 2500 platform. We also show that resulting microbial compositions of mock community samples are not significantly altered when amplicon libraries are prepared using the 2-step library preparation method and sequenced on the Illumina HiSeq platform. We therefore conclude that while the 2-step PCR preparation method combined with the Illumina HiSeq 2500 platform is preferred, data generated by 1-step or 2-step PCR and sequenced on the Illumina MiSeq or HiSeq 2500 platform can be combined to successfully obtain meaningful conclusions about the environment and sample types of interest (given that the same region is targeted).

### Limitations.

The method is extremely high throughput, and as such may not be suitable for small projects unless these are combined with other samples. Producing a large number of samples ready for pooling requires automation so that time from sample collection to data generation is still reasonable. Overall, automation is required, and this approach might be suitable for microbiome service cores where faster turnaround is needed and running many MiSeq runs is not a viable option because of potential batch effects.

## MATERIALS AND METHODS

### Overall study design.

First, to determine if the choice of library preparation method improved amplification of low-biomass samples, we specifically processed 92 vaginal samples using the dual-indexing 1-step (1) and 2-step (described below) library preparation methods. The success of amplifying the 16S rRNA V3-V4 region from genomic DNA was evaluated for each method.

To then determine if the choice of library preparation method or sequencing platform affected the observed sample microbial composition of these samples, we sequenced the libraries of samples successfully produced using both 1-step and 2-step methods on the Illumina MiSeq (1-step and 2-step) and HiSeq (2-step only) platforms. The same 2-step library was sequenced on the MiSeq and HiSeq platforms. The compositions of samples for which high-quality data were obtained from all three methods were statistically compared.

To further validate if sequencing platform affected observed microbial compositions, we also produced 10 separate V3-V4 16S rRNA gene amplicon libraries from the ZymoBIOMICS microbial community DNA standard (Zymo Research, Irvine, CA) using the 2-step library preparation method, and we sequenced each library on separate runs of the Illumina HiSeq platform. We compared the microbial compositions of these samples to theoretical values reported by Zymo Research as well as to V3-V4 amplicon libraries of the same standard prepared and sequenced by Zymo Research on the Illumina MiSeq platform (see Text S2 in the supplemental material for library preparation and sequencing methods).

Finally, to compare the sequencing quality and per-sample read statistics (per sample number and quality of reads) produced by the Illumina MiSeq and HiSeq 2500 platforms, amplicon libraries from vaginal samples were produced using the 2-step PCR method and sequenced on both the Illumina MiSeq (276 out of possible 576 samples) and HiSeq 2500 (1,194 out of possible 1,568 samples) platforms. All amplicon libraries targeted the 16S rRNA gene V3-V4 regions from human vaginal samples.

### Genomic DNA extraction.

Clinician-collected midvaginal ESwabs were stored in Amies transport medium (Copan, Murrieta, CA) as previously described (4). The study was approved by the University of Maryland Baltimore and the Johns Hopkins School of Public Health Institutional Review Board. Samples were thawed on ice and vortexed briefly. A 0.5-ml aliquot of the cell suspension was transferred to a FastPrep lysing matrix B (MP Biomedicals, Santa Ana, CA) tube containing 0.5 ml of phosphate-buffered saline (PBS; Invitrogen, Carlsbad, CA). A cell lysis solution containing 5 μl lysozyme (10 mg/ml; EMD Chemicals, Gibbstown, NJ), 13 μl mutanolysin (11,700 U/ml; Sigma-Aldrich, St. Louis, MO), and 3.2 μl lysostaphin (1 mg/ml; Ambi Products, LLC, Lawrence, NY) was added, and samples were incubated at 37°C for 30 min. Ten μl Proteinase K (20 mg/ml; Invitrogen), 50 μl 10% SDS (Sigma-Aldrich, St. Louis, MO), and 2 μl RNase A (10 mg/ml; Invitrogen, Carlsbad, CA) were added, and samples were incubated at 55°C for an additional 45 min. Cells were lysed by mechanical disruption on a FastPrep homogenizer at 6 m/s for 40 s, and the lysate was centrifuged on a Zymo Spin IV column at 7,000 × *g* for 1 min (Zymo Research, Irvine, CA). Lysates were further processed on the QIAsymphony platform using the QS DSP virus/pathogen midi kit (Qiagen, Hilden, Germany) according to the manufacturer’s recommendations. DNA quantification was carried out using the Quant-iT PicoGreen double-stranded DNA assay (Invitrogen).

### Sequencing library construction using 1-step PCR.

Sequencing libraries were constructed by amplifying the 16S rRNA gene V3-V4 regions using the 1-step PCR amplification protocol previously described (1). Primer sequences ranged from 90 to 97 bp depending on the length of the heterogeneity spacer (Table 3). Amplification was performed using Phusion *Taq* master mix (1×; ThermoFisher, Waltham, MA) with 3% dimethylsulfoxide (DMSO), 0.4 μM each primer, and 5 μl of genomic DNA. A standard volume of genomic DNA was used for each library, because genomic DNA concentration was not indicative of the number of 16S rRNA gene targets (Fig. S1). Cycling conditions were initial denaturation at 98°C for 30 s, 30 cycles of denaturation at 98°C for 15 s, annealing at 58°C for 15 s, and elongation at 72°C for 15 s, followed by a final elongation step at 72°C for 60 s.

**TABLE 3:** 1-Step PCR primers^*a*^

Primer	Sequence (5′→3′)
Forward	AATGATACGGCGACCACCGAGATCTACAC + GTGACTGGAGTTCAGACGTGTGCTCTTCCGATCT + index (8 bp) + heterogeneity spacer (0–7 bp) + ACTCCTRCGGGAGGCAGCAG
Reverse	CAAGCAGAAGACGGCATACGAGAT + ACACTCTTTCCCTACACGACGCTCTTCCGATCT + index (8 bp) + heterogeneity spacer (0–7 bp) + GGACTACHVGGGTWTCTAAT

a Primer sequences are presented as Illumina MiSeq 3′ flow cell linker + Illumina 5′ sequencing primer (CS1/CS2) + index + heterogeneity spacer + 16S rRNA gene V3-V4 primer.

10.1128/mSystems.00029-19.1 FIG S1 The concentration of genomic DNA extracted from vaginal samples does not correlate to the absolute bacterial load as measured by qPCR of panbacterial 16S rRNA genes. Download FIG S1, PDF file, 0.2 MB. Copyright © 2019 Holm et al. 2019 Holm et al. This content is distributed under the terms of the Creative Commons Attribution 4.0 International license.

### Sequencing library construction using 2-step PCR.

The following library preparation method is a modified version of a method provided by Illumina (https://support.illumina.com/downloads/16s_metagenomic_sequencing_library_preparation.html). The V3-V4 regions of the 16S rRNA genes were targeted from genomic DNA using bacterial primers 338F and 806R, combined with a heterogeneity spacer of 0 to 7 bp and the Illumina sequencing primers (Table 4, step 1). A single PCR master mix containing an equal ratio of all primers, which vary by the length of the heterogeneity spacer, was used for all samples. This strategy reduces any amplification biases that may be introduced by the differing lengths of the heterogeneity spacers and is efficient because the primers do not contain barcode indices (Fig. 1). Each PCR contained 1× Phusion *Taq* master mix (ThermoFisher), step 1 forward and reverse primers (0.4 μM each; Data Set S1), 3% DMSO, and 5 μl of genomic DNA. This standard volume of genomic DNA was used for each library, because genomic DNA concentration was not indicative of the number of 16S rRNA gene targets (Fig. S1). PCR amplification was performed using the following cycling conditions: an initial denaturation at 94°C for 3 min, 20 cycles of denaturation at 94°C for 30 s, annealing at 58°C for 30 s, elongation at 72°C for 1 min, and a final elongation step at 72°C for 7 min. We used only 20 PCR cycles, because biases in microbial community profiles have been reported with higher numbers of cycles (2). The resultant amplicons were diluted 1:20, and 1 μl was used in the second step of PCR. This second amplification step introduces an 8-bp dual-index barcode to the 16S rRNA gene V3-V4 region amplicons (Data Set S1), as well as the flow-cell linker adaptors using primers containing a sequence that anneals to the Illumina sequencing primer sequence introduced in step 1 (Table 4, step 2; see Data Set S1 for full oligonucleotide sequences). Each primer was added to a final concentration of 0.4 μM in each sample-specific reaction, along with Phusion *Taq* master mix (1×) and 3% DMSO. Phusion *Taq* polymerase (ThermoFisher) was used with the following cycling conditions: an initial denaturation at 94°C for 30 s, 10 cycles consisting of denaturation at 94°C for 30 s, annealing at 58°C for 30 s, elongation at 72°C for 60 s, and then a final elongation step at 72°C for 5 min (Fig. 1).

**TABLE 4:** 2-Step protocol PCR primers^*a*^

Primer	Sequence (5′→3′)
Step 1^*a*^	
Forward	ACACTGACGACATGGTTCTACA + heterogeneity spacer (0–7 bp) + ACTCCTRCGGGAGGCAGCAG
Reverse	TACGGTAGCAGAGACTTGGTCT + heterogeneity spacer (0–7 bp) + GGACTACHVGGGTWTCTAAT
Step 2^*b*^	Illumina 3′ flowcell linker + index + CS1/CS2
Forward	AATGATACGGCGACCACCGAGATCTACAC + index (8 bp) + ACACTGACGACATGGTTCTACA
Reverse	CAAGCAGAAGACGGCATACGAGAT + index (8 bp) + TACGGTAGCAGAGACTTGGTCT

a See Data Set S1 for full oligonucleotide sequences. For step 1, primer sequences are presented as Illumina 5′ sequencing primer (CS1/CS2) + heterogeneity spacer + 16S rRNA gene V3-V4 primer.

b See Data Set S1 for full forward and reverse oligonucleotides, respectively. For step 2, primer sequences are presented as Illumina 3′ flow cell linker + index + CS1/CS2.

### Amplicon library pooling for sequencing.

For a large number of samples, library purification and quantification for each sample would be time- and labor-intensive. To streamline the process, we visualized libraries on 2% agarose E-Gel (ThermoFisher) and determined the relative amplification success at the expected ∼627-bp band size (amplicon plus spacer plus all primer sequences plus linker). Strong, clear bands indicate successful amplification, a weak or fuzzy band indicates intermediate success, and no band indicates low amplification success. We then standardized the volume of each sample to pool to either 5, 10, or 15 µl depending on the high, intermediate, or low amplification success of that sample, respectively (Fig. S2 shows a labeled gel example). The pooled samples were cleaned up with AMPure XP (Agencourt/Beckman Coulter, Brea, CA) beads by following the manufacturer’s instructions, and size was selected around 600 bp. After size selection, the DNA was eluted in water. To ensure the proper size of the PCR product, the pooled libraries were run on an Agilent TapeStation 2200 with a DNA1000 tape for quality assurance.

10.1128/mSystems.00029-19.2 FIG S2 Amplicon libraries are run on a 2% E-Gel to determine amplification success and the volume of each library to be pooled for sequencing. Strong, crisp bands are successful amplifications for which 5 μl is used in pooling (green 5). For samples in which no band is observed, 20 μl is used in pooling (red 20). Finally, 10 μl is used in pooling (yellow 10) from samples which produce weaker, fuzzy bands. Download FIG S2, PDF file, 0.5 MB. Copyright © 2019 Holm et al. 2019 Holm et al. This content is distributed under the terms of the Creative Commons Attribution 4.0 International license.

Pooled libraries prepared by 1-step PCR were sequenced on the Illumina MiSeq platform, and those prepared by 2-step PCR were sequenced on both the Illumina HiSeq and MiSeq platforms by following the procedures outlined above.

### Amplification success of vaginal samples using the 1-step and 2-step PCR library preparation methods.

To determine the success or failure of amplifying the 16S rRNA gene V3-V4 regions from vaginal samples, including samples with low absolute bacterial load using the 1-step or 2-step protocols, we evaluated the presence or absence of an amplicon band using agarose gel electrophoresis after the final amplification (in the case of the 2-step protocol, after the 2nd step). Estimates of absolute bacterial abundance in vaginal samples were determined using real-time quantitative PCR as previously described (14). Figure S2 contains an example electrophoresis gel labeled with the volume of the library used for pooling. Samples labeled 20 show no bands, and in this analysis they represent a failure of amplification. All other samples represent successful amplifications.

### Sequencing by Illumina MiSeq and sequence data processing.

Libraries were sequenced on an Illumina MiSeq instrument using 600 cycles, producing 2× 300-bp paired-end reads. The sequences were demultiplexed using the dual-barcode strategy, a mapping file linking barcode to samples and split_libraries.py, a QIIME-dependent script (15). The resulting forward and reverse fastq files were split by sample using the QIIME-dependent script split_sequence_file_on_sample_ids.py, and primer sequences were removed using TagCleaner (version 0.16) (16). Further processing followed the DADA2 workflow for Big Data and DADA2 (v. 1.5.2) (https://benjjneb.github.io/dada2/bigdata.html) (17) (Text S1).

10.1128/mSystems.00029-19.8 TEXT S1 Code used for processing and quality filtering the amplicon sequence data. Download Text S1, TXT file, 0.02 MB. Copyright © 2019 Holm et al. 2019 Holm et al. This content is distributed under the terms of the Creative Commons Attribution 4.0 International license.

10.1128/mSystems.00029-19.9 TEXT S2 Methods from ZymoBIOMICS on the production of the ZymoBIOMICS microbial DNA standard. Download Text S2, PDF file, 0.2 MB. Copyright © 2019 Holm et al. 2019 Holm et al. This content is distributed under the terms of the Creative Commons Attribution 4.0 International license.

### Sequencing by Illumina HiSeq and sequence data processing.

Libraries were sequenced on an Illumina HiSeq 2500 using Rapid Run chemistry and a 515-nm laser barcode reader (a required accessory) and loaded at 8 pmol with a 20% PhiX library. Paired-end 300-bp reads were obtained using a HiSeq rapid SBS kit, v2 (2× 250 bp, 500-cycle kit), combined with a 2× 50 bp, 100-cycle kit (alternatively, a single 500-bp kit plus 2× 50-bp kits can be used). In the HiSeq Control Software, under the Run Configuration tab, within Flow Cell Setup, the Reagent kit Type was set to “HiSeq Rapid v2” and the Flow Cell Type to “HiSeq Rapid Flow Cell v2.” In the next step, within Recipe, the Index Type was set to “Custom,” the Flow Cell Format to Paired End, and the Cycles set to 301, 8, 8, and 301 for Read 1, Index 1, Index 2, and Read 2, respectively (Fig. S3). Instead of the standard sequencing primers, custom locked nucleic acid primers were used according to the Fluidigm access array user guide, appendices B and C (18) (the Fluidigm system itself is not required). These primers are required for sequencing under the modified conditions, so that the CS1 and CS2 regions can be used as primer binding regions (to produce reads 1 and 2) (Fig. 1). The sequences were demultiplexed using the dual-barcode strategy, a mapping file linking barcode to samples (Data Set S1), and split_libraries.py, a QIIME-dependent script (15). The resulting forward and reverse fastq files were split by sample using the QIIME-dependent script split_sequence_file_on_sample_ids.py, and primer sequences were removed using TagCleaner (version 0.16) (16). Further processing followed the DADA2 workflow for Big Data and DADA2 (v. 1.5.2) (17).

10.1128/mSystems.00029-19.3 FIG S3 HiSeq control software configuration for obtaining 2× 300-bp paired-end reads using the Illumina HiSeq 2500. Download FIG S3, PDF file, 1.1 MB. Copyright © 2019 Holm et al. 2019 Holm et al. This content is distributed under the terms of the Creative Commons Attribution 4.0 International license.

### Intraindividual distance-based bacterial community comparisons of vaginal samples.

Samples successfully amplified using both library preparation methods were used for comparative analyses. The 1-step libraries were sequenced on the Illumina MiSeq platform, and the 2-step libraries were sequenced on both the Illumina MiSeq and HiSeq platforms. Sequences were quality filtered and assembled as described above. To fairly compare the Illumina HiSeq and MiSeq platforms, lengths of 255 bp and 225 bp were chosen for hard trimming of forward and reverse reads, respectively, because these were the lengths at which median quality scores decreased below 20 for the lowest-quality library (Fig. S4; specifically the 2-step MiSeq F and R read quality decreases dramatically at approximately these lengths, so the same lengths were applied to all three methods). Individual reads were further truncated at the base, where a quality score of 2 was observed, and filtered to contain no ambiguous bases. Additionally, the maximum number of expected errors in a read was set to 2. Reads were assembled only if the overlap between forward and reverse reads, which occurs in the conserved region between V3 and V4, was 100% identical. Chimeras for combined runs were removed per the DADA2 protocol. A Kruskal-Wallis test was applied to test if differences in the per-sample quality scores differed among the three methods (R Package, stats; function, kruskal.test). For each of the three quality-filtered data sets, amplicon sequence variants (ASVs) generated by DADA2 were individually taxonomically classified using the RDP naïve Bayesian classifier (19) trained with the SILVA v128 16S rRNA gene sequence database (20). ASVs of major vaginal taxa were assigned species-level annotations using speciateIT (version 2.0), a novel and rapid per-sequence classifier (http://ravel-lab.org/speciateit/), and verified via BLASTn against the NCBI 16S rRNA gene sequence reference database. Read counts for ASVs assigned to the same taxonomy were summed for each sample. To test for differences in the quality scores of samples prepared and sequenced by the different methods, a Kruskal-Wallis rank sum test was applied. To determine if library preparation methods influenced microbial community β-diversity, samples were assigned a vaginal community state type as defined by Jensen-Shannon distances and clustering via Ward linkage (3). Clusters of Jensen-Shannon distances were visualized using t-Stochastic Neighbor Embedding (tSNE) (21) using 5,000 iterations and perplexity set to 30. Agreement of within-subject assigned CSTs between methods was determined using Fleiss’ kappa statistic (22) (R package irr, v 0.84). Here, κ of 0 indicates all CST assignments were dissimilar between the libraries, and κ of 1 indicates identical CST assignments. A κ of >0.75 is considered excellent agreement.

10.1128/mSystems.00029-19.4 FIG S4 Per-cycle quality score summaries for vaginal samples prepared and sequenced using the 1-step PCR method and Illumina MiSeq (*n* = 49 samples), 2-step PCR method and Illumina MiSeq (*n* = 83 samples), and 2-step PCR method and Illumina HiSeq (*n* = 83 samples). Shown for each cycle are the mean quality score (green line), the median quality score (solid orange line), and the quartiles of the quality score distribution (dotted orange lines). Download FIG S4, PDF file, 0.2 MB. Copyright © 2019 Holm et al. 2019 Holm et al. This content is distributed under the terms of the Creative Commons Attribution 4.0 International license.

10.1128/mSystems.00029-19.5 FIG S5 Panbacterial qPCR determination of the number of 16S rRNA gene targets in vaginal samples from women of various ages (left). Vaginal samples which were amplified by 2-step PCR but not 1-step PCR had significantly lower absolute abundances of 16S rRNA gene targets (*U =* 790.5, *P* = 0.027), and subjects from which samples came were significantly older (*U* = 484.5, *P* = 0.001). Download FIG S5, PDF file, 0.2 MB. Copyright © 2019 Holm et al. 2019 Holm et al. This content is distributed under the terms of the Creative Commons Attribution 4.0 International license.

10.1128/mSystems.00029-19.6 FIG S6 (Top) Microbial compositions of the ZymoBIOMICS microbial DNA standard prepared and sequenced using the 2-step library preparation method described in this paper and the Illumina HiSeq platform (2-step_HiSeq) or Zymo prepared and Illumina MiSeq sequenced (Zymo-MiSeq) are statistically similar to the theoretical values reported by Zymo. Taxa in boldface compose the mock communities. (Bottom) Multidimensional scaling plot of Jensen-Shannon distances of the same samples as those at the top. Download FIG S6, PDF file, 0.2 MB. Copyright © 2019 Holm et al. 2019 Holm et al. This content is distributed under the terms of the Creative Commons Attribution 4.0 International license.

10.1128/mSystems.00029-19.7 FIG S7 FastQC output from 3 HiSeq Rapid Run (HiSeq RR) and MiSeq sequencing runs. Download FIG S7, PDF file, 1.3 MB. Copyright © 2019 Holm et al. 2019 Holm et al. This content is distributed under the terms of the Creative Commons Attribution 4.0 International license.

### Comparison of mock community microbial compositions between Illumina HiSeq runs.

We used the ZymoBIOMICS microbial community DNA standard (Zymo Research) as a mock community for this analysis. To maintain consistency in taxonomic annotations, we used BLAST and the NCBI reference database to classify each sequence variant in these analyses. Specific single-nucleotide variants produced different taxonomic classifications in the following taxa due to truncation to the V3-V4 amplicon regions: Bacillus subtilis to Bacillus mojavensis, Listeria monocytogenes to Listeria welshimeri, and Escherichia coli to Escherichia fergusonii; however, we manually verified the identity of the sequence variants. To determine if the mock community amplicon library compositions produced by our 2-step library preparation and HiSeq sequencing methods were within the same variations as those observed by Zymo Research, we statistically compared the distributions of Jensen-Shannon distances between the Zymo MiSeq samples and the reported theoretical values and our HiSeq samples and the theoretical values using a Mann-Whitney-Wilcoxon test (R Package stats; function, wilcox.test).

### Sequencing quality comparisons of Illumina HiSeq and Illumina MiSeq sequencing of 2-step PCR amplicon libraries.

To compare the sequence quality produced on the near-full Illumina MiSeq and HiSeq runs, the per-cycle mean, median, and 1st and 3rd quartiles (Q1 and Q3) were calculated from quality scores of sample-specific forward and reverse fastq files in R, version 3.4.4 (15 March 2018), using the qa function of the ShortRead package, v 1.36.1 (23), data.table, v 1.11.4, and ggplot2, v 3.0.0 (24) (R notebook html available upon request). Because quality scores were not normally distributed, a Mann-Whitney-Wilcoxon test was applied to test if differences in the quality scores per cycle differed between the two sequencing platforms (R Package stats; functions, shapiro.test and wilcox.test).

### Data availability.

All sequence data have been uploaded to the Sequence Read Archive under BioProject number PRJNA489669.
