# Supplementary figures and images for: Ultrahigh-Throughput Multiplexing and Sequencing of >500-Base-Pair Amplicon Regions on the Illumina HiSeq 2500 Platform
Source: mSystems. 2019 Feb 19;4(1):e00029-19. doi: 10.1128/mSystems.00029-19 (PMC6381223; doi:10.1128/mSystems.00029-19)

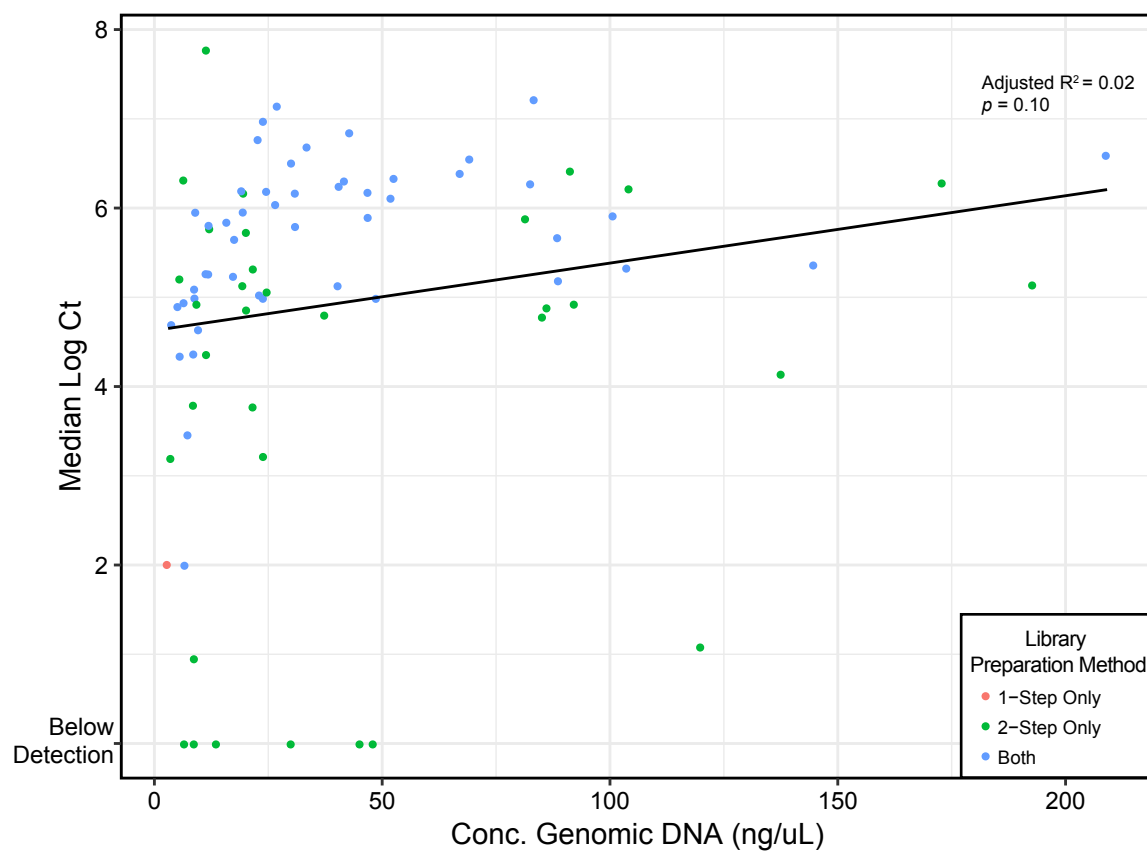

FIG S1

Supplement: FIG S1 [file mSystems.00029-19-sf001.pdf]

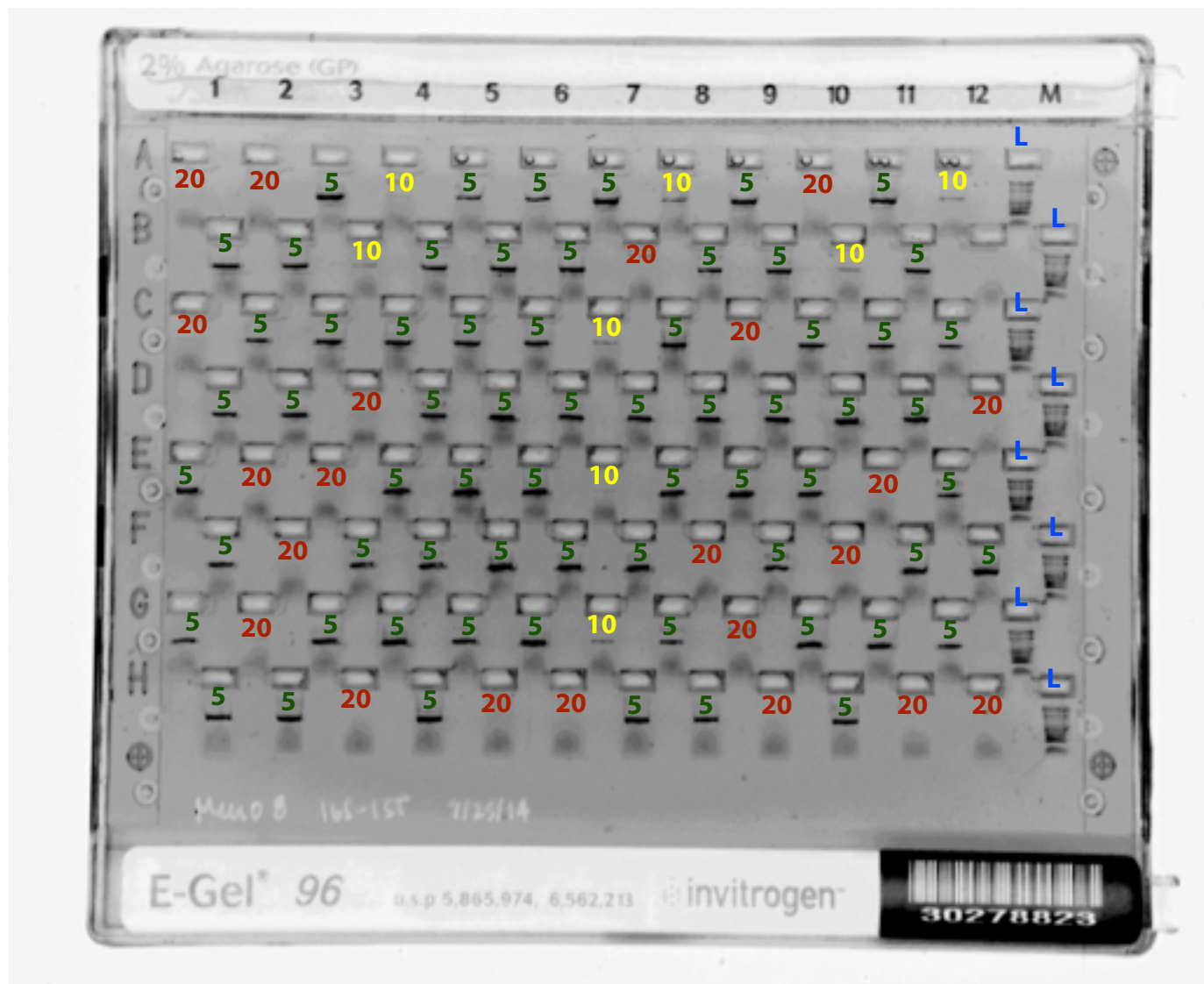

FIG S2

Supplement: FIG S2 [file mSystems.00029-19-sf002.pdf]

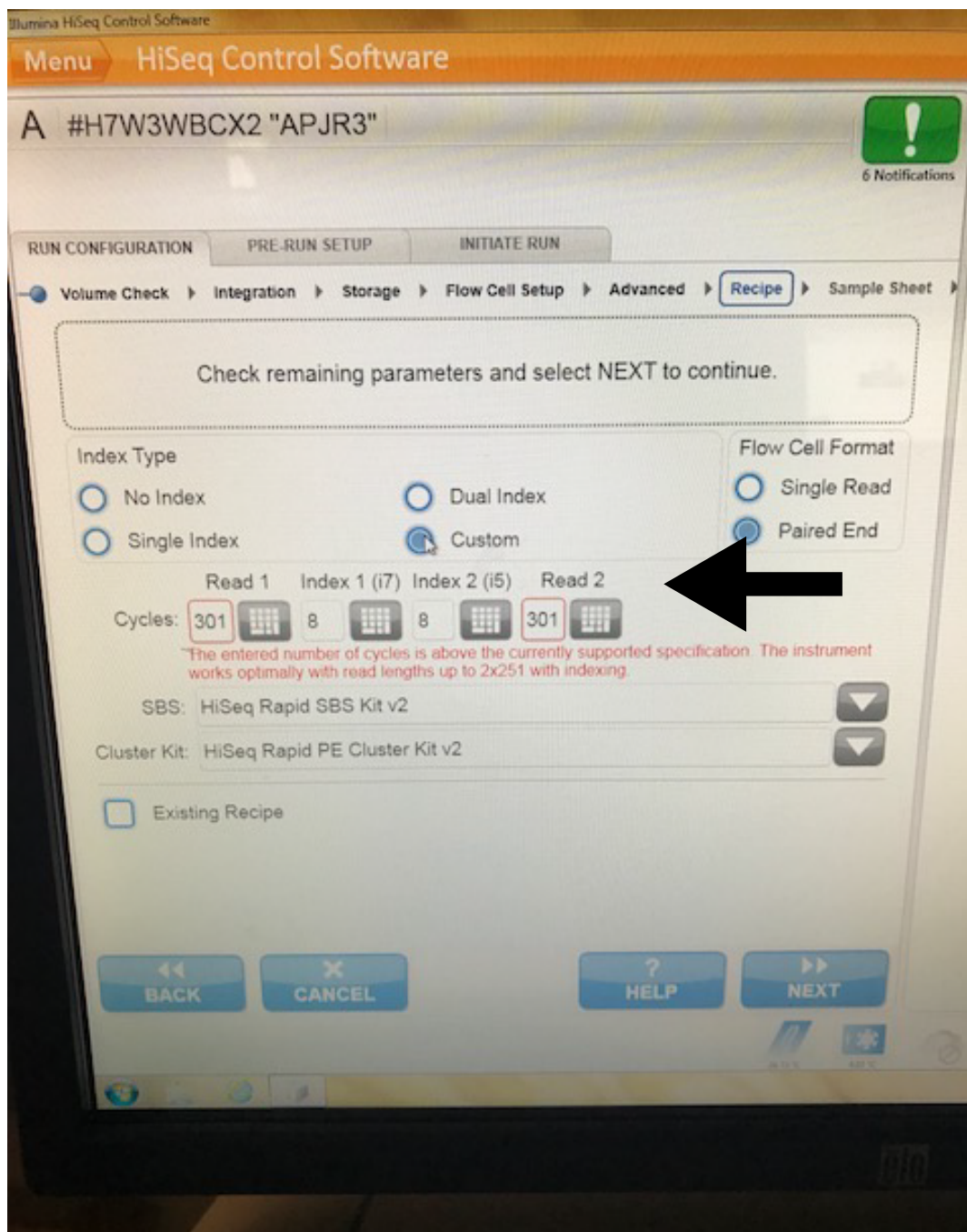

FIG S3

Supplement: FIG S3 [file mSystems.00029-19-sf003.pdf]

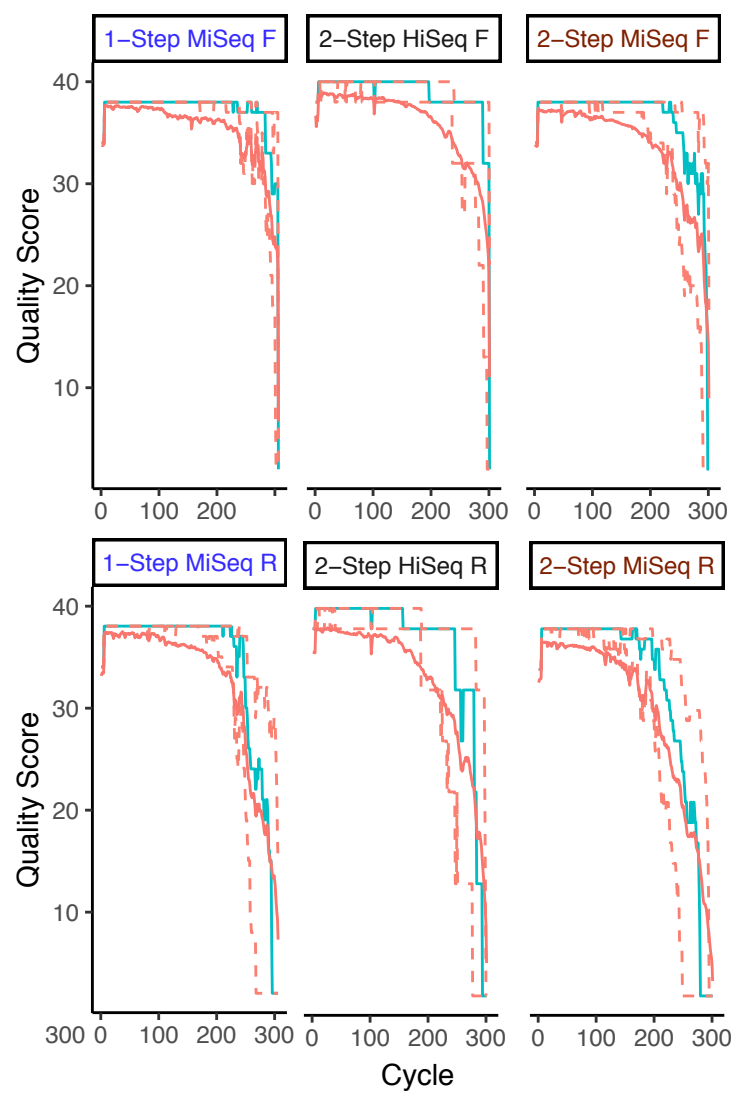

FIG S4

Supplement: FIG S4 [file mSystems.00029-19-sf004.pdf]

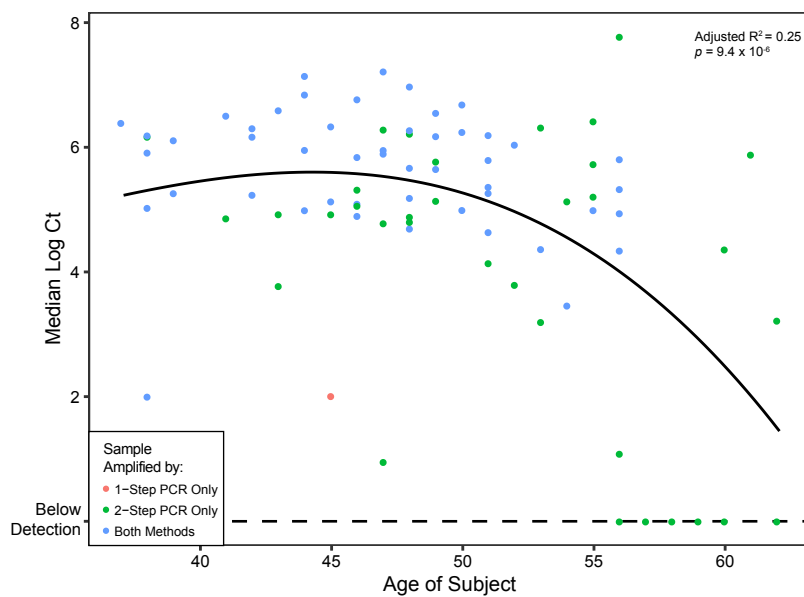

FIG S5

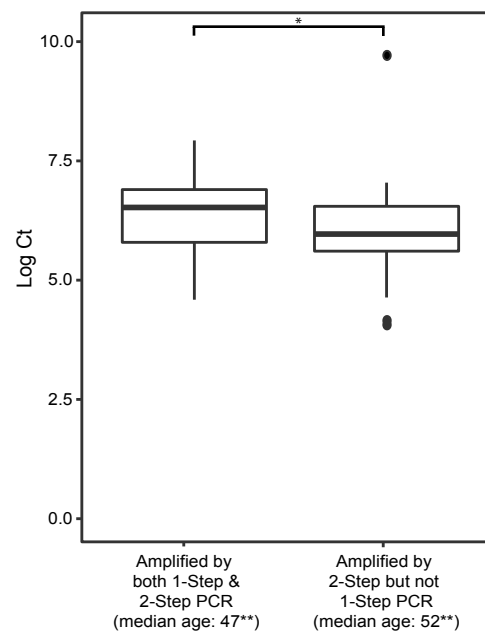

Supplement: FIG S5 [file mSystems.00029-19-sf005.pdf]

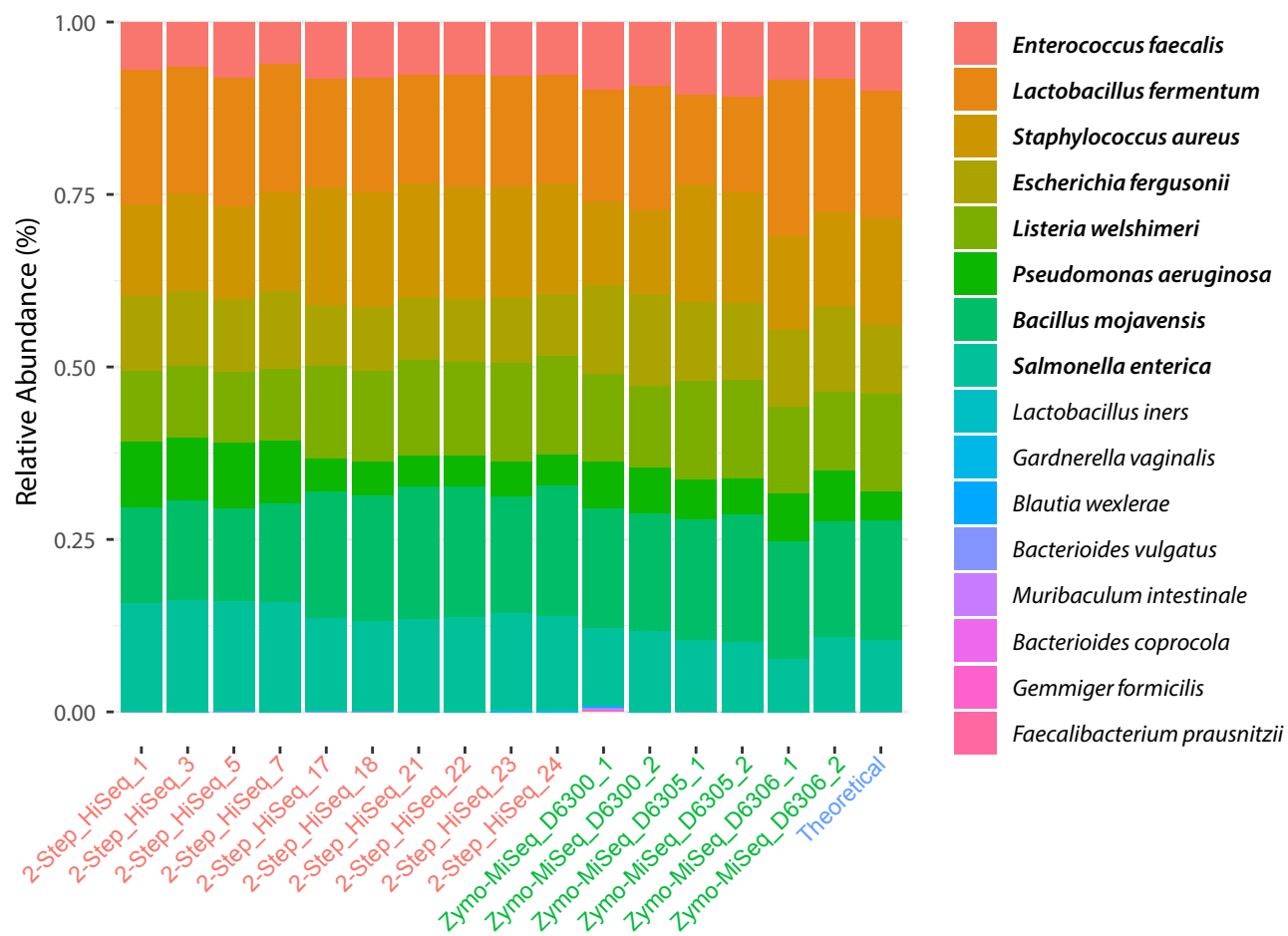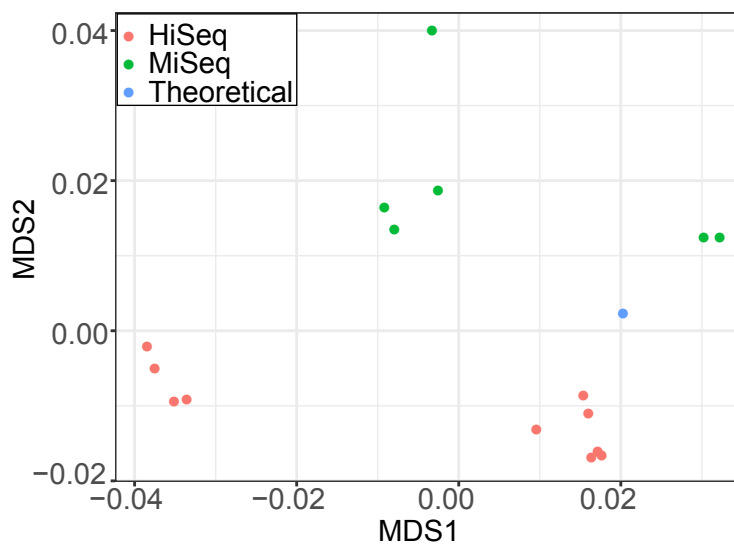

FIG S6

Supplement: FIG S6 [file mSystems.00029-19-sf006.pdf]

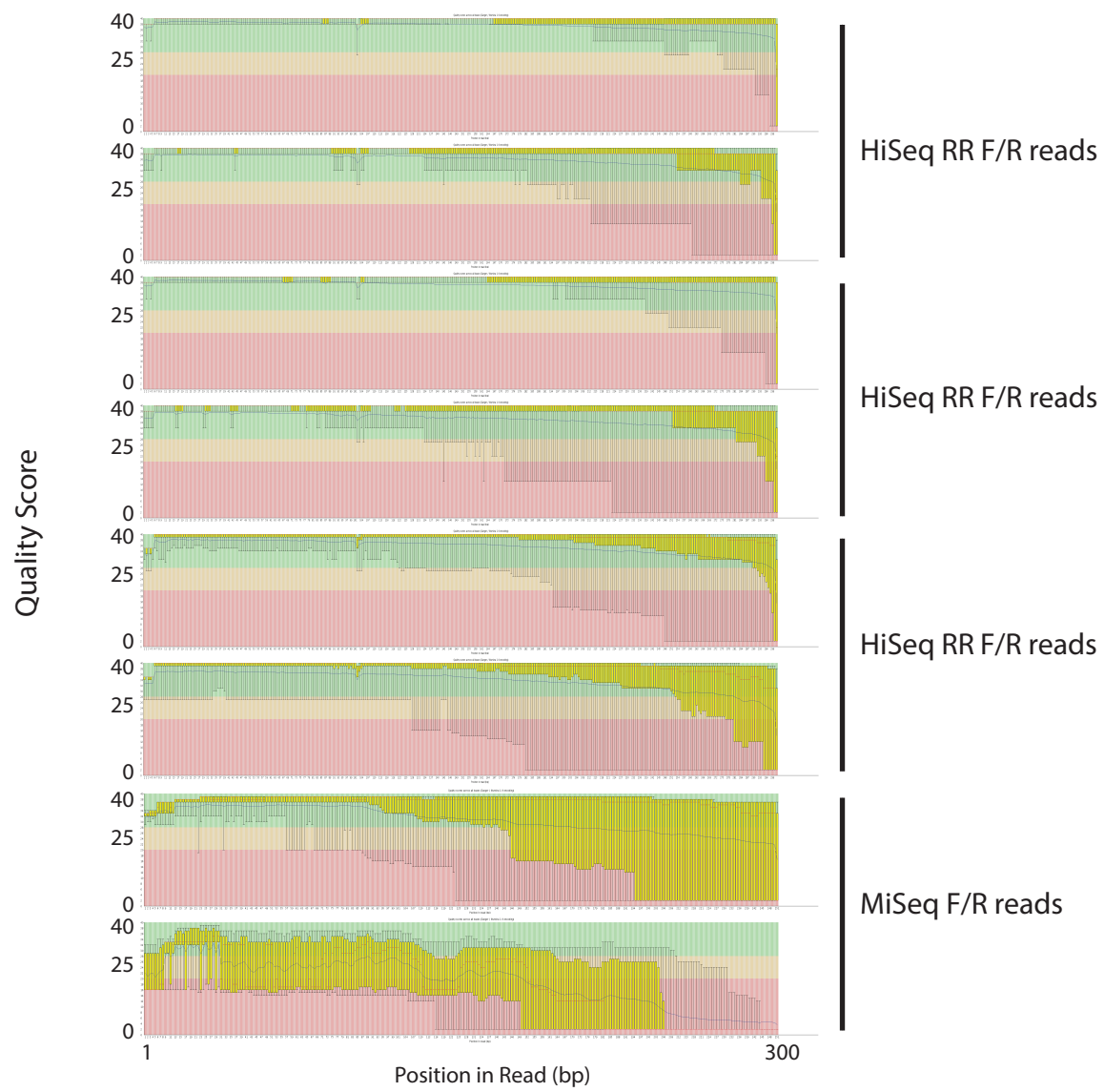

FIG S7

Supplement: FIG S7 [file mSystems.00029-19-sf007.pdf]
